# Microproteins (miPs) – the next big thing

**DOI:** 10.1186/1478-811X-10-42

**Published:** 2012-12-18

**Authors:** Stephan Feller

**Affiliations:** 1 Biological Systems Architecture Group, Department of Oncology, Weatherall Institute of Molecular Medicine, John Radcliffe Hospital, Headley Way, Oxford, OX3 9DS, United Kingdom

## Abstract

With iPS cells, sncRNAs, chromatin modification regulation and cancer stem cells already cooling off again, i.e. not being guaranteed publications in the 'ultimate' journals anymore, what will be very soon the new red-hot (or super-cool, i.e. anything but lukewarm) 'kid on the block'? We would vote for microproteins.

## 

In case you do not know what they are, no need to worry: nobody does.

However, with proto-genes recently entering the stage [1] it seems just a small step for mankind to assume that those dye fronts of protein gels you have been cutting off and binning for decades now harbour a plethora of precious little gems and thus an amazing potential for discovering exciting regulatory molecules that will bind to proteins etc. to regulate their conformations and activities, their binding partners, their subcellular localisation and so on.

They can be expected to steer embryonic development and stem cell differentiation, to play a role in cancers and neurodegenerative diseases and should also make great leads for future drugs. Clearly, miPs are yet another Nobel Prize lurking, and begging for attention.

To study them is easy: just mass spec your protein gel dye fronts to death, synthesise all found miP candidates, biotinylate them and go fishing for binding partners. Then introduce your miPs into cells using cell-penetrating shuttle peptides or transfections and watch their interaction partners misbehave in cells. Finally, do miP knock-outs and -ins in animals of your fancy and observe what happens to them. Alternatively, if you do not have access to mass spec-omics, look for short transcribed ORFs of unknown function in the genome, synthesise those ORFs as biotinylated peptides on a vast scale and then go fishing.

That is all there is to it really,…so…would someone look, PLEASE.

Of course, if you actually find something, we told you so, and we would want to share the glory and the financial rewards; and do not dare to simply call these newly found, super-exciting, sparkling entities merely regulatory peptides or christen them with another boring name like that, or you shall never be grand.

Merry Christmas and a healthy, happy and very productive 2013 to all of you.
